# Randomized evaluation of 5-month Ticagrelor monotherapy after 1-month dual-antiplatelet therapy in patients with acute coronary syndrome treated with drug-coated balloons: REC-CAGEFREE II trial rationale and design

**DOI:** 10.1186/s12872-024-03709-1

**Published:** 2024-01-20

**Authors:** Chao Gao, Bin Zhu, Jianzheng Liu, Zhiwei Jiang, Tao Hu, Qiong Wang, Yi Liu, Ming Yuan, Fei Li, Ruining Zhang, Jielai Xia, Yoshinobu Onuma, Duolao Wang, Patrick Serruys, Ling Tao, on behalf of the REC-CAGEFREE II Study Group

**Affiliations:** 1 Department of Cardiology, Xijing Hospital, Changle West Road, Xi’an, 710032 China; 2 Beijing KeyTech Statistical Consulting Co., Beijing, 100015 China; 3 Department of Statistics, Air Force Medical University, Xi’an, 710000 China; 4 Department of Cardiology, University of Galway, Galway, H91 TK33 Ireland; 5 Biostatistics Unit, Liverpool School of Tropical Medicine, Liverpool, L3 5QA UK

**Keywords:** Drug-coated balloon, Dual antiplatelet therapy, Acute coronary syndrome

## Abstract

**Background:**

Patients treated with drug-coated balloons (DCB) have the theoretical advantage of adopting a low-intensity antiplatelet regimen due to the absence of struts and polymers. Nevertheless, the optimal antiplatelet strategy for patients undergoing DCB-only treatment remains a topic of debate and has not been investigated in randomized trials.

**Methods:**

The REC-CAGEFREE II is an investigator-initiated, prospective, open-label, multi-center, randomized, non-inferiority trial aimed to enroll 1908 patients from ≥ 40 interventional cardiology centers in China to evaluate the non-inferiority of an antiplatelet regimen consisting of Aspirin plus Ticagrelor for one month, followed by five months Ticagrelor monotherapy, and then Aspirin monotherapy for six months (Experimental group) compared to the conventional treatment of Aspirin plus Ticagrelor for 12 months (Reference group) in patients with acute coronary syndrome (ACS) who have undergone percutaneous coronary intervention (PCI) using paclitaxel-coated balloons (DCB) exclusively. Participants will be randomly assigned to the Experimental or Reference group in a 1:1 ratio. The randomization will be stratified based on the center and the type of lesion being treated (De novo or in-stent restenosis). The primary endpoint is net adverse clinical events (NACE) within 12 months of PCI, which includes the composite of all-cause death, any stroke, any myocardial infarction, any revascularization and Bleeding Academic Research Consortium (BARC) defined type 3 or 5 bleeding. The secondary endpoint, any ischemic and bleeding event, which includes all-cause death, any stroke, MI, BARC-defined type 3 bleeding, any revascularization, and BARC-defined type 2 bleeding events, will be treated as having hierarchical clinical importance in the above order and analyzed using the win ratio method.

**Discussion:**

The ongoing REC-CAGEFREE II trial aims to assess the efficacy and safety of a low-intensity antiplatelet approach among ACS patients with DCB. If non-inferiority is shown, the novel antiplatelet approach could provide an alternative treatment for ACS patients with DCB.

**Trial registration:**

ClinicalTrials.gov identifier: NCT04971356.

**Supplementary Information:**

The online version contains supplementary material available at 10.1186/s12872-024-03709-1.

## Background

Drug-coated balloons (DCB) have emerged as a promising therapeutic option for the management of coronary atherosclerotic heart disease (CAD) by percutaneous coronary intervention (PCI). Currently, the management of ISR by DCB is considered a Class IA recommendation [1]. The safety and effectiveness of the DCB-only strategy have also been studied and demonstrated in de novo small vessels [2], acute coronary syndromes (ACS) [3, 4], and high-bleeding risk patients [5]. Furthermore, the application of DCB is gradually expanding to include de novo large vessels [6, 7].

Bleeding after PCI remains a substantial clinical challenge. Clinical evidence has indicated that the occurrence of major bleeding after PCI is associated with a 5.7-fold increase in the risk of mortality [8]. The administration of antiplatelet medications is a major contributing factor to bleeding events following PCI. The conventional approach of dual antiplatelet therapy (DAPT) for patients with ACS undergoing PCI involves using Aspirin in combination with a potent P2Y12 inhibitor for 12 months [9]. While this approach effectively reduces the risk of ischemic events, it also exposes patients to a considerable risk of bleeding. To address this issue, alternative antiplatelet strategies, such as the abbreviation of DAPT duration [10, 11], have been investigated for reducing bleeding after drug-eluting stent (DES) implantation. For high-bleeding risk patients, the abbreviated DAPT approach has been recommended in the guidelines of the European Society of Cardiology (ESC) and the American College of Cardiology/American Heart Association/Society for Cardiovascular Angiography and Interventions (ACC/AHA/SCAI) [9, 12].

Patients who receive exclusive treatment with DCB may have the theoretical advantage of adopting a low-intensity antiplatelet regimen [13] because of the strut and polymer-free nature of DCB, which could result in a lower thrombotic burden and inflammation than DES [14, 15]. However, despite extensive research on the optimal antiplatelet strategy for patients treated with DES [16–21], there is currently a lack of randomized data specifically investigating the optimal DAPT regimen for DCB-treated patients.

To fill the knowledge gap, we designed the REC-CAGEFREE II trial aimed to investigate the potential non-inferiority of a treatment regimen consisting of one month of dual antiplatelet therapy (DAPT) followed by five months of Ticagrelor monotherapy and then Aspirin monotherapy for six months, compared to the conventional DAPT for 12 months, in patients with ACS who have undergone PCI with DCB exclusively.

## Study design

### Objectives and hypothesis

**Fig. 1:**
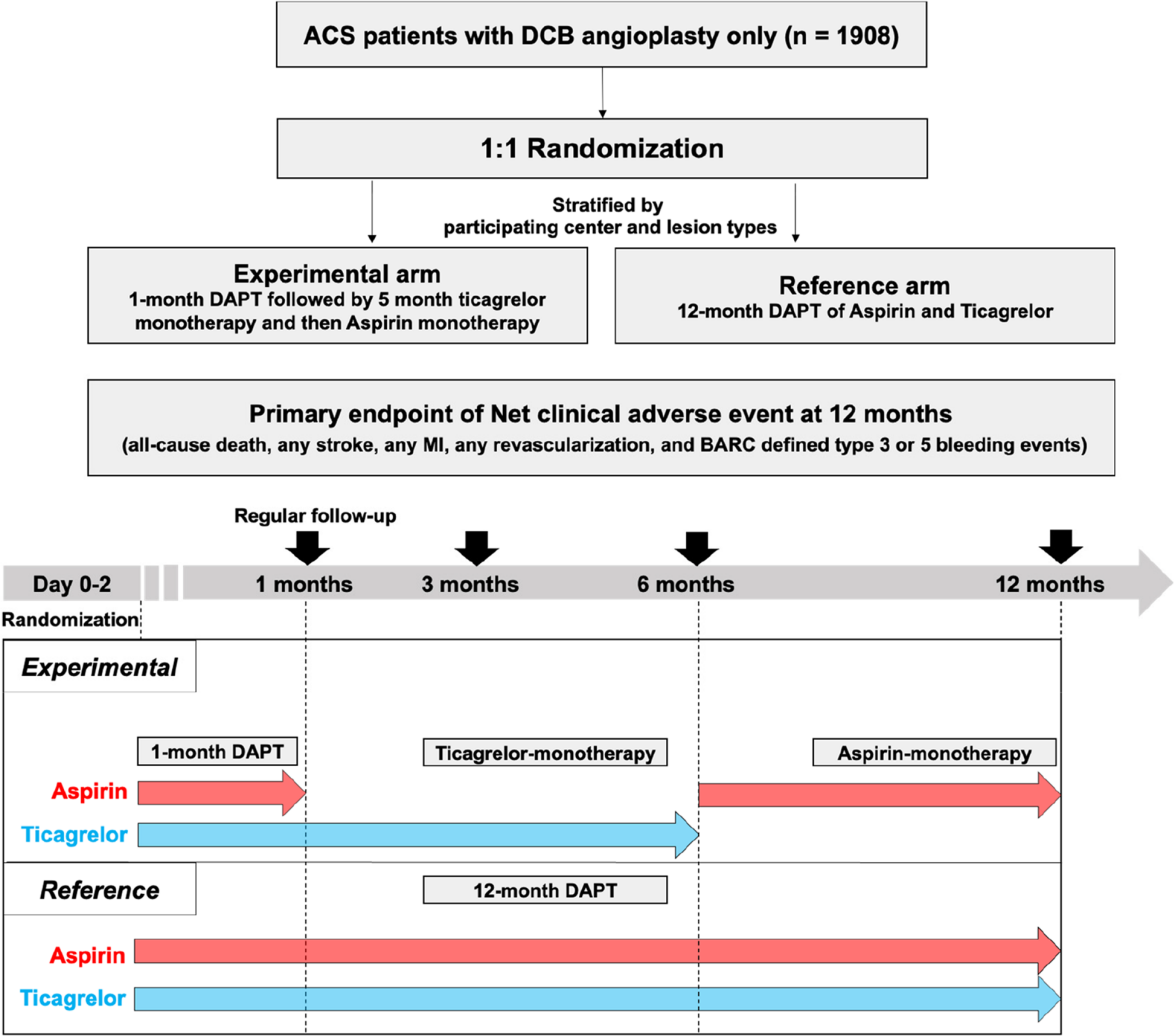
Schematic study design and flow diagram

The REC-CAGEFREE II trial (ClinicalTrials.gov identifier: NCT04971356) is an investigator-initiated, multicenter, prospective, randomized, open-label trial aimed to enroll 1908 patients from ≥ 40 interventional cardiology centers in China. The primary objective of the trial is to test the non-inferiority of a treatment regimen consisting of Aspirin plus Ticagrelor for one month followed by five months of Ticagrelor monotherapy, and finally, Aspirin monotherapy for six months (Experimental group), in comparison to Aspirin plus Ticagrelor for 12 months (Reference group) (Fig. 1) in ACS patients treated exclusively with paclitaxel-coated balloon. The incidence of Net adverse clinical events (NACE) at 12 months will be assessed as the primary endpoint to determine the overall risk in clinical practice considering both ischemic and bleeding adverse events. The secondary objective is to assess if the Experimental group is superior to the Reference group with regard to the incidence of any ischemic and bleeding endpoints, including all-cause death, any stroke, MI, BARC-defined type 3 bleeding, any revascularization, and BARC-defined type 2 bleeding events (in this hierarchy), analyzed using a win ratio method.

### Study organization and funding

This trial is investigator-initiated and received unrestricted grant support from Yinyi Biotech (Liaoning, China). Yinyi Biotech had no product portfolio in any antiplatelet medication. Apart from this sponsorship, Yinyi Biotech had no involvement in the design, execution, or decision to publish the study. The steering committee has a pivotal role with overall responsibility for the concept, design, and execution of the study progress in accordance with scientific, medical, ethical, and practical elements. The committee will convene a meeting to ensure the effective management and execution of the study, including data acquisition, security, analysis, and reporting. The study follows the ethical principles outlined in the Declaration of Helsinki and has received approval from the institutional review board at each participating center for its protocol.

### Study population

Patients who have undergone paclitaxel-coated balloon angioplasty for the treatment of ACS will be screened for eligibility to participate in the study. ACS includes unstable angina, defined as typical symptoms, including recurrent episodes at rest or at minimal effort, with transient ST-segment elevation or depression or angiographic visual diameter stenosis ≥ 90%, plaque rupture, or thrombotic lesions. MI is defined as the presence of clinical symptoms, electrocardiographic changes, or abnormal imaging findings of MI combined with an increase in creatine kinase myocardial band fraction above the upper normal limit or an increase in troponin-T or troponin-I to greater than the 99th percentile of the upper normal limit [1, 22].

The specific brand, length, or diameter of the paclitaxel-coated balloon used during the procedure was determined at the investigators' discretion. Both de novo and in-stent restenosis lesions are included. Inclusion and exclusion criteria are outlined in Table 1. Once eligible patients provide voluntary informed consent, the allocation of treatment and implementation of study procedures, including baseline measurements, will commence. To ensure that eligible patients fully comprehend the purpose and procedures of the investigation without encountering any language barriers, the study may opt to enroll patients of Chinese nationality and ethnicity exclusively.

**Table 1:** Inclusion and exclusion criteria

**Inclusion criteria**
1. Patients with an indication for PCI due to acute coronary syndrome (including STEMI, NSTEMI, and unstable angina)
2. All intended target lesion(s) are successfully treated by PCI with only drug-coated balloon(s)
3. Patients who are able to complete the follow-up and compliant to the prescribed medication
**Exclusion criteria**
1. Under the age of 18 or older than 80 years old
2. Unable to give informed consent
3. Patient is a woman who is pregnant or nursing
4. Known contraindications to medications such as heparin, antiplatelet drugs, or contrast
5. Currently participating in another trial and not yet at its primary endpoint
6. Planned elective surgery
7. Concurrent medical condition with a life expectancy of less than 1 years
8. Previous intracranial hemorrhage
9. Required long-term oral anticoagulant therapy
10. Cardiogenic shock
11. Previous stent implantation within 6 months
12. In-stent thrombosis
13. Target lesion located in surgical conduit

*PCI* percutaneous coronary intervention, *ACS* acute coronary syndrome

The treatment of DCB should adhere to the recommendations of the German Consensus Group on DCB interventions [23] and the Third Report of the International DCB Consensus Group [24]. Additional information on the recommendations of DCB angioplasty can be found in the Supplemental material. Patients who undergo bailout stenting after DCB or receive a combination treatment of DCB and DES will be ineligible to participate in this trial.

Investigators may exercise discretion in utilizing antithrombotic medications, glycoprotein IIb/IIIa inhibitors, intravascular imaging, or fractional flow reserve. Complete revascularization in one PCI session is recommended. If a staged procedure becomes necessary, it will be documented during the initial procedure, and the patient will continue to be in the screening phase until the staged procedure is finalized. Randomization will be conducted after the completion of the staged procedure. If stents are inserted for any reason during the staged procedure, the patient will be considered a screening failure, disqualifying them from participating in the trial.

### Randomization

Randomization and assignment will occur within 48 h post-PCI. Patients will be randomized in a 1:1 fashion to the Experimental arm, which involves DAPT for one month followed by five months of Ticagrelor monotherapy, and then Aspirin monotherapy for six months, or the Reference arm, which entails the DAPT for 12 months (Fig. 1). All DAPT treatments will consist of a combination of Aspirin and Ticagrelor. Web-response dynamic-block randomization, utilizing mixed blocks of 2 or 4, will allocate random assignment stratified by center and the type of lesion being treated (De novo or in-stent restenosis).

Loading doses of Aspirin 300 mg and Ticagrelor 180 mg will be administered if the patient is not taking Aspirin or Ticagrelor at the time of PCI. For daily maintenance, patients will be prescribed Aspirin 100 mg Q.D and Ticagrelor 90 mg B.I.D. Concomitant use of other antiplatelet agents or anticoagulants will not be permitted. While the physician has discretion over other medical treatments, it is strongly advised to implement guideline-directed medical therapy to address the patient's specific condition, such as controlling hypertension or diabetes mellitus, prescribing high-intensity statins, discontinuing cigarette smoking, and providing optimal pharmacologic treatment for heart failure.

Patients who have been prescribed Clopidogrel before PCI will be switched to Ticagrelor as soon as possible after randomization. It is recommended to substitute Clopidogrel with Ticagrelor at the next scheduled medication administration. Irrespective of the timing and dosage of the previous Clopidogrel regimen, Ticagrelor will be prescribed with a loading dose of 180 mg in accordance with the recommendation [25]. In case a patient experiences dyspnea and is unable to continue taking Ticagrelor, they can be substituted with Clopidogrel. These patients will still be included in the study and will not be considered as major protocol deviations. The replacement of Ticagrelor with Clopidogrel should be carried out in accordance with consensus [25]. Detailed instructions on how to switch between oral P2Y12 inhibitors can be found in Supplementary Fig. 1.

### Follow-up

Scheduled follow-up visits occur at 1 (± 14 days), 3, 6, and 12 (± 30 days) months post-randomization. All follow-up visits are preferably scheduled on-site. If the patients are unable or unwilling to visit the outpatient clinic, the scheduled visit can be replaced by a telephone call except for the 30-day and one-year visits. At each visit, self-reported adherence to study and non-study medications are collected together with the assessment of any cardiac or cerebrovascular ischemic or bleeding occurrences or any serious adverse event. The WeChat account of each participant will be documented for record-keeping purposes. To facilitate the acquisition of patient-reported outcomes and adherence to the allocated medications, we developed a mobile application that functions through the WeChat platform. The participants will be contacted monthly through this application. All participants will be contacted monthly through this application and receive a questionnaire to evaluate their health status and adherence.

### Study endpoints

The study endpoints are listed in Table 2. The primary endpoint is the NACE within 12 months of PCI. NACE is defined as a composite endpoint of all-cause death, any stroke, any myocardial infarction (MI), any revascularization, and BARC-defined type 3 or 5 bleeding events. Briefly, all-cause deaths will be considered cardiac unless an undisputed non-cardiac cause is present. The definition of Academic Research Consortium (ARC)-2 will be followed [26]. Stroke is defined as any non-convulsive focal or global neurological deficit of abrupt onset lasting for more than 24 h or leading to death, which is caused by ischemia or hemorrhage within the brain. The Neuro-ARC definition and classification will be used [27]. MI will be defined according to the fourth universal definition of myocardial infarction [22] and a dedicated sensitivity analysis will be implemented by defining MI using other criteria [26, 28, 29]. Revascularization will be determined according to the ARC-2 criteria [26]. Bleeding will be defined by the Bleeding Academic Research Consortium (BARC) criteria [30], and other definitions [31–35] will used for exploratory purposes. Routine follow-up angiography in the absence of symptoms was not recommended. The adherence to the medication allocated will be assessed according to Non-adherence Academic Research Consortium (NARC) [36] definitions.

**Table 2:** Study endpoints

**Primary efficacy endpoint**
Net adverse clinical events (NACE), defined as a composite clinical endpoint of all-cause death, any stroke, any MI, any revascularization, and BARC-defined type 3 or 5 bleeding events (Time Frame: 12 months)
**Primary Safety Endpoint**
Patient-oriented composite endpoint (PoCE), defined as a composite clinical endpoint of all-cause death, any stroke, any MI, any revascularization (Time Frame: 12 months)
**Secondary endpoints**
1. Any ischemic or bleeding event, including any death, any stroke, any MI, BARC-defined type 3 bleeding events, any revascularization, and BARC type 2 bleeding events, will be treated as having hierarchical clinical importance and analyzed using the win ratio method (Time Frame: 12 months)
2. NACE (Time Frame: 1 and 6 months)
***Bleeding endpoints***
3. BARC defined type 3 or 5 bleeding events (Time Frame: 1, 6, and 12 months)
4. BARC defined type 2, 3 or 5 bleeding events (Time Frame: 1, 6, and 12 months)
5. BARC defined type 2 bleeding events (Time Frame: 1 and 12 months)
***Ischemic endpoints***
6. POCE (Time Frame: 1, 6, and 12 months)
7. Individual components of PoCE (Time Frame: 1, 6, and 12 months)
8. Device-oriented Composite Endpoint (DoCE) (Time Frame: 1, 6, and 12 months)
*DoCE is a composite clinical endpoint of cardiac cause death, target vessel myocardial infraction (TV-MI), and Clinically indicated target lesion revascularization (CI-TLR)*
9. Individual components of DoCE (Time Frame: 1, 6, and 12 months)
10. Target vessel failure (TVF) (Time Frame: 1, 6, and 12 months)
*Target vessel failure is defined as cardiovascular death, target vessel myocardial infraction (TV-MI), and clinically-indicated target vessel revascularization*
11. Clinical indicated target vessel revascularization (Time Frame: 1, 6, and 12 months)
12. Definite/Probable Stent thrombosis rates according to ARC-II classification (Time Frame: 1, 6, and 12 months)

*NACE* net adverse clinical events, *MI* myocardial infarction, *BARC* Bleeding Academic Research Consortium, *SCAI* Society for Cardiovascular Angiography and Interventions, *FUD* Fourth Universal Definition, *DoCE* Device-oriented Composite Endpoint, *TV-MI* target vessel myocardial infarction, *CI-TLR* clinically indicated target lesion revascularization, *PoCE* patient-oriented composite endpoint, *TVF* Target vessel failure, *CI-TVR* clinically indicated target vessel revascularization, *ST* stent thrombosis, *ARC* Academic Research Consortium

Suspected adverse events, including bleeding and ischemic outcomes, were reported promptly on an electronic case report form, with source documents centrally collected. After collecting adverse events centrally, any record that could lead to the unblinding of treatment assignment was obliterated before submission to the clinical event committee. All adverse events were categorized according to predefined criteria by an independent clinical-event adjudication committee (CEC) whose members were unaware of the assignment group.

#### Sample size calculations

This study compares Experimental and Reference treatments at the individual patient level. Our hypothesis is that in patients with ACS who undergo PCI using DCB exclusively, a treatment regimen consisting of Aspirin plus Ticagrelor for one month, followed by five months of Ticagrelor monotherapy, and finally, Aspirin monotherapy for six months, would be non-inferior to conventional 12-month DAPT treatment in terms of overall ischemic and bleeding risks as determined by the rate of NACE.

Due to the limited availability of data on the occurrence rate of NACE in ACS patients treated with DCB, the event rate of the Reference group in this trial was estimated by referring to the findings of the GLOBAL LEADERS [37, 38] and TICO [20] trials, which was made under the consideration that patients treated with DES would have a comparable or lower risk of experiencing an ischemic event compared to those treated with DCB. Additionally, it was assumed that both DCB and DES-treated patients would have a similar risk of bleeding if they were administered a similar DAPT regimen. Therefore, it is anticipated that 8% of patients in the Reference treatment arm will reach the primary endpoint at one year. The non-inferiority margin of 3.2%, which was 40% of the NACE rate, was chosen based on clinically acceptable relevance according to the margins in previous major trials comparing antiplatelet regimens after DES implantation [16, 17, 39, 40] and the feasibility of patient enrolment. With a total of 1812 patients (906 per group), the study is estimated to have 80% power to show non-inferiority with a 5% one-sided α error rate [16, 40, 41]. Accounting for an attrition rate of approximately 5%, the final sample size was determined to be at least 1908 patients (954 per group).

#### Statistical considerations

The demographic and clinical variables at baseline will be summarized for each treatment group, considering both the intention-to-treat (ITT) and per-protocol (PP) populations. Categorical data will be described as numbers (percentages). Continuous variables will be expressed as mean ± standard deviation or median (interquartile range) for normal or skewed distributions.

The primary endpoint of the trial is NACE at 12 months after randomization. The primary analysis will be based on a crude measurement of treatment difference in the primary endpoint, without adjusting for any covariates, using the intention-to-treat (ITT) population. To estimate the cumulative event rate of NACE at 12 months in each group, the Kaplan–Meier (KM) method will be employed. The one-sided 95% confidence interval (CI) for the difference in the cumulative event rate at 12 months between the Experimental group and the Reference group will be calculated using Greenwood's formula for the variance of the KM estimates. If the upper limit of the one-sided 95% CI is below 3.2%, it will be concluded that the Experimental group is non-inferior to the Reference group. In addition, a covariate-adjusted analysis of the primary endpoint, considering the covariates at baseline, lesion characteristics (ISR or de novo), and center effect, will be performed as a sensitivity analysis. Additional information on covariate-adjusted analysis can be found in the Supplemental material. The crude and adjusted analyses will be repeated in the per-protocol population to support the primary results.

The secondary endpoint, any ischemic or bleeding events, will be treated as having hierarchical clinical importance and analyzed using the win ratio method in the order of all-cause death, any stroke, MI, BARC-defined type 3 bleeding, any revascularization and BARC-defined type 2 bleeding events. For other secondary endpoints, the difference in cumulative event rate and their 95%CIs will be reported, and Cox proportional hazard ratios (HR) will also be provided.

To maintain overall alpha for primary and secondary endpoints at 12 months, a hierarchical sequential testing structure will be implemented. Supplementary Table 1 presents the fixed sequence to be followed [42–44]. The prespecified subgroup analyses will also be conducted for clinically relevant factors such as age, sex, body mass index, diabetes mellitus or smoking, and other risk indicators, with details described in Supplementary Table 2. Stratum-specific HRs and corresponding 95% CI will be calculated for each subgroup using a Cox proportional hazards model. Interaction testing will be performed using the subgroup X treatment allocation as an additional term in the Cox model. A prespecified landmark analysis of the primary endpoint will also be performed from 1 to 12 months since treatment during the first month is the same in both groups. Unless otherwise specified, a two-sided test will be utilized for testing at a 5% significance level.

#### Safety monitoring

The Data and Safety Monitoring Board (DSMB), in conjunction with the steering committee responsible for ensuring participant safety, will act in an advisory capacity to monitor participant safety, evaluate the study progress, and review procedures for maintaining data confidentiality. A biannual DSMB meeting will be held, either in-person or via teleconference, to discuss study progress, ensure proper execution of study procedures, maintain data quality and security, and review any safety concerns related to participants. Although no interim analysis was initially planned, the DSMB holds the power to terminate the study process and scrutinize relevant events during the trial in the event of any safety issues.

## Discussion

The incidence of ischemic events is at its highest during the first month after PCI and tends to decrease thereafter [11]. By contrast, the risk of bleeding with DAPT, despite being relatively high in the first few days after PCI due to the use of an arterial access site and periprocedural antithrombotic therapy, does not diminish over time when antiplatelet therapy is continued. Consequently, the overall benefit of the conventional 12-month DAPT regimen may diminish over time, particularly for ACS patients who received DAPT with Ticagrelor, which strongly blocks ADP and also has an effect on thromboxane-mediated platelet activation [45], and Aspirin plays a minimal role in platelet inhibition in the presence of strong P2Y12 receptor blockage [46].

Approaches of shortening the course of DAPT in ACS patients with DES have been investigated [39, 40, 47]. However, this approach is controversial due to the fact that studies have indicated that opting for a 3-month or 6-month DAPT, as opposed to the conventional 12-month DAPT, is associated with a higher risk of MI [40] or associated with higher rates of mortality and stent thrombosis [47].

In recent years, the treatment strategy of DAPT [10] de-escalation or abbreviation [11, 16–20, 48] has been widely investigated and shown to be associated with favorable clinical outcomes. The TWILIGHT trial [18, 49] showed that among high-risk patients who underwent PCI and completed three months of DAPT, Ticagrelor monotherapy was associated with a lower incidence of clinically relevant bleeding than Ticagrelor plus Aspirin, without increasing the risk of ischemic events. In addition, the benefits of ticagrelor monotherapy concerning bleeding events were more pronounced in patients with ACS [49]. The TICO study [20] suggested that among patients with ACS treated with DES, Ticagrelor monotherapy after three months of DAPT resulted in a modest but statistically significant reduction in a composite outcome of major bleeding and cardiovascular events at one year. In addition, although the superiority of the Experimental regimen with one month of DAPT followed by Ticagrelor monotherapy was not determined in the GLOBAL LEADERS trial, its greater benefit in lowering bleeding risk was also demonstrated among ACS subgroups [38]. In contrast, a clopidogrel monotherapy-based de-escalation of DAPT after 1 to 2 months failed to attest noninferiority to 12 months of DAPT for the net clinical benefit [50].

Compared with the DES, due to the absence of a metallic scaffold and polymer inside the coronary artery and the effective retention period of antiproliferative drugs being only 1 to 3 months, the use of DCB might associated with faster vessel healing and lower thrombotic burden. However, there is no randomized data investigating how this difference between DCB and DES could be reflected in the course and intensity of the antiplatelet strategy.

Based on empirical experiences and non-randomized data, in 2013, the consensus from the German group [23] suggested for ACS patients with DCB, the recommended duration of DAPT is 12 months. The consensus of DAPT developed by the European Society of Cardiology (ESC) in 2017 stated that in patients treated with DCB, dedicated clinical trials investigating the optimal duration of DAPT are lacking. As a result, the use of DAPT in DCB-treated patients was recommended to be the same as in DES-treated patients [25]. The latest ESC guideline for ACS in 2023 [9] and the ACC/AHA/SCAI guideline for Coronary Artery Revascularization in 2021 [12] still do not provide a recommendation for the DAPT regimen after DCB.

In contrast to the blooming evidence that emerged during the past five years investigating the optimal antiplatelet regimens post-PCI for patients with DES, so far, clinical evidence of the use of antiplatelet medications in DCB-treated patients is scarce, and consequently, the use of antiplatelet strategies in those patients are diverse. Up to July 2021, among the 34 RCTs comparing DES and DCB in the ACS or ACS plus CCS population, there were notable differences in the recommendations for the duration of DAPT in the DCB arm. Seven studies suggested one month of DAPT, ten studies recommended three months, six studies utilized six months, and ten studies adopted 12 months of DAPT (Supplementary Table 3).

The current REC-CAGEFREE II trial will provide novel and clinically meaningful insights into the potential role of a low-intense antiplatelet regimen in ACS patients after PCI with DCB. Findings from this trial may have major implications regarding the necessity of short DAPT followed by Ticagrelor monotherapy for six months and Aspirin monotherapy thereafter in ACS patients treated with DCB, and thereby offer a novel antiplatelet strategy to simultaneously lower bleeding risk while maintaining anti-ischemic efficacy.

### Current status of the REC-CAGEFREE II trial

The REC-CAGEFREE II trial enrolled the first patient in November 2021 and the last patient in March 2023; a total of 1948 patients were finally enrolled at the 41 participating sites. The completion of the study follow-up is projected for March 2024, and the trial results are expected to be reported in the third quarter of 2024.

## Summary

The ongoing REC-CAGEFREE II trial aims to assess the efficacy and safety of a low-intensity antiplatelet approach among ACS patients with DCB. If non-inferiority is shown, the novel antiplatelet approach could provide an alternative treatment for ACS patients with DCB.

### Supplementary Information

**Additional file 1: Supplemental material. Supplementary Table 1.** Multiplicity considerations and hierarchical testing of the primary and secondary endpoints. **Supplementary Table 2.** Planned Subgroup analyses. **Supplementary Table 3.** Antiplatelet used in the DCB arm in RCTs comparing POBA/BMS/DES to DCB (Up to July 2021 or Up to Oct 2023). **Supplementary Figure 1.** Switching between oral P2Y12 [46].

## Data Availability

No datasets were generated or analysed during the current study.
