# Supplementary material for: Randomized evaluation of 5-month Ticagrelor monotherapy after 1-month dual-antiplatelet therapy in patients with acute coronary syndrome treated with drug-coated balloons: REC-CAGEFREE II trial rationale and design
Source: BMC Cardiovasc Disord. 2024 Jan 20;24:62. doi: 10.1186/s12872-024-03709-1 (PMC10799378; doi:10.1186/s12872-024-03709-1)
Supplement: Supplementary file 1 — Additional file 1: Supplemental material. Supplementary Table 1. Multiplicity considerations and hierarchical testing of the primary and secondary endpoints. Supplementary Table 2. Planned Subgroup analyses. Supplementary Table 3. Antiplatelet used in the DCB arm in RCTs comparing POBA/BMS/DES to DCB (Up to July 2021 or Up to Oct 2023). Supplementary Figure 1. Switching between oral P2Y12 [46]. [file 12872_2024_3709_MOESM1_ESM.docx]

**Supplemental material**

**Randomized evaluation of 5-month Ticagrelor monotherapy after 1-month dual-antiplatelet therapy in patients with acute coronary syndrome treated with drug-coated balloons: REC-CAGEFREE II trial** **rationale and design**

This supplementary material has been provided by the authors to give readers additional information about their work.

**Study design supplements**

***The recommendation of DCB angioplasty***

The treatment of DCB should adhere to the recommendations of the German Consensus Group on DCB interventions [1] and the Third Report of the International DCB Consensus Group [2].

With or without a Plain Old Balloon Angioplasty (POBA), a pre-dilation prior to DCB angioplasty shall be performed with a noncompliant balloon, cutting balloon, scoring balloon, or NSE balloon at 0.8-1.0 balloon/vessel size ratio. After lesion preparation, a 10-minute observational period should be conducted, followed by an angiogram to ensure satisfactory lesion preparation, which consists of the following criteria: 1) ≤30% residual stenosis (visual); 2) Thrombolysis In Myocardial Infarction (TIMI) flow grade 3; and 3) the absence of a flow-limiting dissection. The DCB should only be used after successful pre-dilatation. Subsequently, the DCB, on each side longer than the DCB by at least 2-3 mm to avoid geographical mismatch, is inflated at nominal pressure for 30-45 seconds. In cases where subjects experience TIMI flow<3, severe dissection (type D, E, and F), or visual residual stenosis>30% (visual) post-DCB, a bailout DES is recommended to be implanted for rescue treatment. Patients with any bailout stent will be disqualified from participating in this trial.

***Covariate adjusted analysis***

Adjusted analyses will be carried out on the primary outcome to determine whether the treatment effect estimate is affected by the inclusion of covariates at baseline. The covariates that will be included in the adjusted analyses are age (continuous), sex (binary), lesion characteristics (binary: ISR, de novo), hypertension (binary), hyperlipidemia (binary), diabetes (binary), smoking status (categorical), history of CVD (binary), stroke (binary), and clinical presentation (categorical: STEMI, NSTEMI, UA).

We will first calculate a propensity score with treatment as the dependent variable (1 for the experimental group and 0 for the reference group), and all covariates listed above as independent variables through a logistic regression model with a random effect by recruitment centers, and then perform an Inverse Probability Treatment Weighting (IPTW) analysis (weighted KM method). The adjusted difference in NACE rate between the two groups and its one-sided 95%CI will be estimated. This analysis will be performed as ancillary to the primary endpoint analysis.

**Supplementary Table 1. Multiplicity considerations and hierarchical testing of the primary and secondary endpoints**

The primary hypothesis is to analyze the non-inferiority of the NACE rates at 12 months between experimental and reference groups. A hierarchical (sequential) testing structure will be used to maintain overall alpha for secondary endpoints at 12 months, and no additional multiplicity adjustment to the hypothesis testing will be needed.

This structure follows the fixed sequence procedure. If the primary endpoint is statistically significant, the remaining secondary endpoints will be tested in the following order: Non-inferiority testing of the primary endpoint -> Superiority testing of the secondary endpoints in the order of the following:

- The occurrence of any ischemic or bleeding event, including all-cause death, any stroke, MI, BARC-defined type 3 bleeding, any revascularization, and BARC-defined type 2 bleeding events at 12 months (Analyzed by Win Ratio)
- The occurrence of BARC type 2, 3, or 5 bleeding events with the cumulative event rate calculated at 12 months
- The occurrence of BARC type 3 or 5 bleeding events with the cumulative event rate calculated at 12 months
- The occurrence of BARC 2 bleeding events with the cumulative event rate calculated at 12 months
- The occurrence of BARC 3 bleeding events with the cumulative event rate calculated at 12 months
- The occurrence of BARC 5 bleeding events with the cumulative event rate calculated at 12 months

If the test fails to reject the null hypothesis at a 5% significance level, the hierarchical sequential testing will stop, otherwise carry on to the next test, and family-wise type I error will not be inflated.

Analyses of other secondary outcomes and additional analyses for the primary outcome are regarded as exploratory in nature, therefore, multiplicity adjustment will not be applied.

**Supplementary Table 2. Planned Subgroup analyses**

| Concomitant disease and baseline risk factors |
| --- |
| 1. Age <75 years vs. Age≥75 years 2. Male vs. Female 3. BMI <25 kg/m2 vs. BMI≥25 kg/m2 4. Hypertension patients vs. non-hypertension patients 5. Hyperlipidemia patients vs. non-hyperlipidemia patients 6. Diabetic patients vs. non-diabetic patients 7. Renal insufficiency patients vs. non-renal insufficiency patients 8. Baseline smoking vs. non-smoking 9. COPD patients vs. non-COPD patients 10. PVD patients vs. non-PVD patients 11. Previous cardiovascular disease patients vs. non-previous cardiovascular disease patients 12. Previous stroke or TIA patients vs. non-previous stroke or TIA patients 13. LVEF% <50 vs. ≥50 14. Baseline WBC level (median) 15. Baseline platelets level (median) 16. Impact of bleeding risks (according to PRECISE-DAPT, DAPT, CRUSADE, ACUITY, PARIS score or according to ARC definition) and their interaction with treatment 17. Impact of SYNTAX score and its derived scores (functional SYNTAX score, SYNTAX Score II, residual SYNTAX score, logistic clinical SYNTAX Score) in risk stratifying ischemic risks or the complexity of PCI and its interaction with treatment 18. Clinical presentation (STEMI, NSTEMI, UA) 19. Baseline CHF vs. without CHF 20. Baseline heart rate (median) |
| PCI procedure |
| 1. Effect of procedural centers on prognosis 2. In-Stent Restenosis vs. De novo 3. Pre-dilatation balloon types (Non-compliant balloons, cutting or scoring balloons) 4. Single-vessel disease vs. multivessel disease 5. Long lesions ≥28 mm vs. short lesions <28 mm 6. Bifurcation lesions vs. others 7. Impact of DS% pre and post PCI 8. Impact of QFR/FFR/iFR pre and post PCI 9. Radial access vs. femoral access 10. Complex PCI vs. non-complex PCI 11. Proximal LAD vs. non-Proximal LAD 12. Device diameter < 3.0mm vs ≥3.0mm 13. Impact of staged PCI |
| Medication |
| 1. Optimal medical treatment (OMT) specified in the guidelines 2. ACE-I/ARNI 3. SGLT-2 4. Statin/PCSK-9 inhibitor 5. Insulin 6. Proton Pump Inhibitor 7. Impact of adherence to antiplatelet therapy |
| COVID-19 |
| 1. Impact of COVID-19 |

BMI, body mass index; COPD, chronic obstructive pulmonary disease; PVD, peripheral vascular disease; TIA, transient ischemic attack; LVEF, left ventricular ejection fraction; WBC, white blood cell count; ARC, Academic Research Consortium; DAPT, dual antiplatelet therapy; CHF, chronic heart failure; CRP, C-reactive protein; PCI, percutaneous coronary intervention; ISR, in stent restenosis; QFR, quantitative ﬂow ratio; FFR, Fractional Flow Reserve; iFR, instantaneous wave-free ratio; IVUS, intravenous ultrasound; OCT, optical coherence tomography; ACEI, angiotensin-converting enzyme inhibitor; ARNI, angiotensin receptor-neprilysin inhibitor; SGLT-2: sodium-glucose cotransporter-2; PCSK-9, proprotein convertase subtilisin/kexin type 9; NSAID, non-steroidal anti-inflammatory drugs.

**Supplementary Table 3** **Antiplatelet used in the DCB arm in RCTs comparing POBA/BMS/DES to DCB (Up to July 2021)**

|  | **1M** | **3M** | **6M** | **12M** | **Unknown** |
| --- | --- | --- | --- | --- | --- |
| **ACS+CCS** |  | **TIS [3]** | **ISAR DESIRE III [4]** | **DARE [5]** |  |
|  |  |  | **ISAR DESIRE IV [6]** |  |  |
| **NSTEMI + UA + CCS** | **RESTORE ISR China [7]** |  | **RESTORE [8]** | *BASKET-SMALL 2 [9]* |  |
|  | *DEBUT [10]* |  | *RESTORE SVD [11]* | *PICCOLETO II [12]* |  |
|  | *Shin et al. [13]* |  |  |  |  |
| **UA+CCS** | **PACOCATH ISR I [14]** | **PEPCAD II [15]** | **PEPCAD-DES [16]** | **PEPCAD CHINA ISR [17]** | **BIOLUX [18]** |
|  | **PACOCATH ISR II [19]** | **RIBS V [20]** |  | **Ali et al. [21]** |  |
|  | *PICCOLETO [22]* | **RIBS IV [23]** |  | **ELEGANT [24]** |  |
|  | *BELLO [25]* | **Habara et al. [26]** |  | *BEYOND [27]* |  |
|  |  | **Zhu et al. [28]** |  |  |  |
|  |  | **Hu et al. [29]** |  |  |  |
|  |  | *DEBIUT [30]* |  |  |  |
|  |  | **Hamm et al. [31]** |  |  |  |
|  |  | *Funatsu et al. [32]* |  |  |  |
|  |  | *Ali et al. [33]* |  |  |  |
|  |  |  |  |  |  |
| **NSTEMI or STEMI** |  |  | *Hao et al. [34]* | *REVELATION [35]* |  |
|  |  |  |  | *Gobic et al. [36]* |  |
|  |  |  |  | *PEPCAD- NSTEMI [37]* |  |
| **Total** | **7** | **10** | **6** | **10** | **1** |

***italics*= studies of de novo lesion**

**Bold= studies of ISR lesion**

**Antiplatelet used in the DCB arm in RCTs comparing POBA/BMS/DES to DCB (Up to Oct 2023)**

|  | **1M** | **3M** | **6M** | **12M** | **Unknown** |
| --- | --- | --- | --- | --- | --- |
| **ACS+CCS** |  | **TIS [3]** | **ISAR DESIRE III [4]** | **DARE [5]** |  |
|  |  |  | **ISAR DESIRE IV [6]** | *Yu et al. [38]* |  |
|  |  |  |  | *PEPCAD China SVD [39]* |  |
| **NSTEMI + UA + CCS** | **RESTORE ISR China [7]** |  | **RESTORE [8]** | *BASKET-SMALL 2 [9]* |  |
|  | *DEBUT [10]* |  | *RESTORE SVD [11]* | *PICCOLETO II [12]* |  |
|  | *Shin et al. [13]* |  |  |  |  |
| **UA+CCS** | **PACOCATH ISR I [14]** | **PEPCAD II [15]** | **PEPCAD-DES [16]** | **PEPCAD CHINA ISR [17]** | **BIOLUX [18]** |
|  | **PACOCATH ISR II [19]** | **RIBS V [20]** |  | **Ali et al. [21]** |  |
|  | *PICCOLETO [22]* | **RIBS IV [23]** |  | **ELEGANT [24]** |  |
|  | *BELLO [25]* | **Habara et al. [26]** |  | *BEYOND [27]* |  |
|  | **Dissolve ISR [40]** | **Zhu et al. [28]** |  | *Ahmad et al. [41]* |  |
|  |  | **Hu et al. [42]** |  | *BIO-RISE CHINA [43]* |  |
|  |  | *DEBIUT ^[30]^* |  |  |  |
|  |  | **Hamm et al. [31]** |  |  |  |
|  |  | *Funatsu et al. ^[32]^* |  |  |  |
|  |  | *Ali et al. [33]* |  |  |  |
|  |  | *AGENT JAPAN [44]* |  |  |  |
| **NSTEMI or STEMI** |  | *Wang et al. [45]* | *Hao et al. [34]* | *REVELATION [35]* |  |
|  |  |  |  | *Gobic et al. [36]* |  |
|  |  |  |  | *PEPCAD*  *NSTEMI [37]* |  |
| **Total** | **8** | **13** | **6** | **14** | **1** |

***italics*= studies of de novo lesion**

**Bold= studies of ISR lesion**

**Supplementary Figure 1** **Switching between oral P2Y12**[46]

**A. Switching between oral agents in the acute/early phase.**

In the acute/early phase (≤30 days from the index event), switching should occur with the administration of a loading dose (LD) in most cases, with the exception of patients who are de-escalating therapy because of bleeding or bleeding concerns, in whom a maintenance dose (MD) of Clopidogrel (C) should be considered. Timing of switching should be 24 hours after the last dose of a given drug, with the exception of when escalating to Ticagrelor (T), when the LD can be given regardless of the timing and dosing of the previous Clopidogrel regimen.

*Consider de-escalation with Clopidogrel 75-mg MD (24 hours after last Ticagrelor dose) in patients with bleeding or bleeding concerns.

**B. Switching between oral agents in the late/very late phase.**

In the late/very late phase (>30 days from the index event), switching should occur with the administration of an MD 24 hours after the last dose of a given drug, with the exception of patients changing from Ticagrelor therapy, for whom an LD should be considered. De-escalation from Ticagrelor should occur with administration of an LD 24 hours after the last dose of Ticagrelor (but in patients in whom de-escalation occurs because of bleeding or bleeding concerns, an MD of Clopidogrel should be considered). *Consider de-escalation with Clopidogrel 75-mg MD (24 hours after last Ticagrelor dose) in patients with bleeding or bleeding concerns.

**Reference**

1. Kleber FX, Rittger H, Bonaventura K, Zeymer U, Wöhrle J, Jeger R, Levenson B, Möbius-Winkler S, Bruch L, Fischer D *et al*: **Drug-coated balloons for treatment of coronary artery disease: updated recommendations from a consensus group**. *Clinical research in cardiology : official journal of the German Cardiac Society* 2013, **102**(11):785-797.

2. Jeger RV, Eccleshall S, Wan Ahmad WA, Ge J, Poerner TC, Shin ES, Alfonso F, Latib A, Ong PJ, Rissanen TT *et al*: **Drug-Coated Balloons for Coronary Artery Disease: Third Report of the International DCB Consensus Group**. *JACC Cardiovascular interventions* 2020, **13**(12):1391-1402.

3. Pleva L, Kukla P, Kusnierova P, Zapletalova J, Hlinomaz O: **Comparison of the Efficacy of Paclitaxel-Eluting Balloon Catheters and Everolimus-Eluting Stents in the Treatment of Coronary In-Stent Restenosis: The Treatment of In-Stent Restenosis Study**. *Circulation Cardiovascular interventions* 2016, **9**(4):e003316.

4. Byrne RA, Neumann FJ, Mehilli J, Pinieck S, Wolff B, Tiroch K, Schulz S, Fusaro M, Ott I, Ibrahim T *et al*: **Paclitaxel-eluting balloons, paclitaxel-eluting stents, and balloon angioplasty in patients with restenosis after implantation of a drug-eluting stent (ISAR-DESIRE 3): a randomised, open-label trial**. *Lancet* 2013, **381**(9865):461-467.

5. Baan J, Jr., Claessen BE, Dijk KB, Vendrik J, van der Schaaf RJ, Meuwissen M, van Royen N, Gosselink ATM, van Wely MH, Dirkali A *et al*: **A Randomized Comparison of Paclitaxel-Eluting Balloon Versus Everolimus-Eluting Stent for the Treatment of Any In-Stent Restenosis: The DARE Trial**. *JACC Cardiovascular interventions* 2018, **11**(3):275-283.

6. Kufner S, Joner M, Schneider S, Tölg R, Zrenner B, Repp J, Starkmann A, Xhepa E, Ibrahim T, Cassese S *et al*: **Neointimal Modification With Scoring Balloon and Efficacy of Drug-Coated Balloon Therapy in Patients With Restenosis in Drug-Eluting Coronary Stents: A Randomized Controlled Trial**. *JACC Cardiovascular interventions* 2017, **10**(13):1332-1340.

7. Chen Y, Gao L, Qin Q, Chen S, Zhang J, Chen H, Wang L, Jin Z, Zheng Y, Zhang Z *et al*: **Comparison of 2 Different Drug-Coated Balloons in In-Stent Restenosis: The RESTORE ISR China Randomized Trial**. *JACC Cardiovascular interventions* 2018, **11**(23):2368-2377.

8. Wong YTA, Kang DY, Lee JB, Rha SW, Hong YJ, Shin ES, Her SH, Nam CW, Chung WY, Kim MH *et al*: **Comparison of drug-eluting stents and drug-coated balloon for the treatment of drug-eluting coronary stent restenosis: A randomized RESTORE trial**. *American heart journal* 2018, **197**:35-42.

9. Jeger RV, Farah A, Ohlow MA, Mangner N, Möbius-Winkler S, Leibundgut G, Weilenmann D, Wöhrle J, Richter S, Schreiber M *et al*: **Drug-coated balloons for small coronary artery disease (BASKET-SMALL 2): an open-label randomised non-inferiority trial**. *Lancet* 2018, **392**(10150):849-856.

10. Rissanen TT, Uskela S, Eränen J, Mäntylä P, Olli A, Romppanen H, Siljander A, Pietilä M, Minkkinen MJ, Tervo J *et al*: **Drug-coated balloon for treatment of de-novo coronary artery lesions in patients with high bleeding risk (DEBUT): a single-blind, randomised, non-inferiority trial**. *Lancet (London, England)* 2019, **394**(10194):230-239.

11. Tang Y, Qiao S, Su X, Chen Y, Jin Z, Chen H, Xu B, Kong X, Pang W, Liu Y *et al*: **Drug-Coated Balloon Versus Drug-Eluting Stent for Small-Vessel Disease: The RESTORE SVD China Randomized Trial**. *JACC Cardiovasc Interv* 2018, **11**(23):2381-2392.

12. Cortese B, Di Palma G, Guimaraes MG, Piraino D, Orrego PS, Buccheri D, Rivero F, Perotto A, Zambelli G, Alfonso F: **Drug-Coated Balloon Versus Drug-Eluting Stent for Small Coronary Vessel Disease: PICCOLETO II Randomized Clinical Trial**. *JACC Cardiovasc Interv* 2020, **13**(24):2840-2849.

13. Shin ES, Lee JM, Her AY, Chung JH, Eun Lee K, Garg S, Nam CW, Doh JH, Koo BK: **Prospective randomized trial of paclitaxel-coated balloon versus bare-metal stent in high bleeding risk patients with de novo coronary artery lesions**. *Coronary artery disease* 2019, **30**(6):425-431.

14. Scheller B, Hehrlein C, Bocksch W, Rutsch W, Haghi D, Dietz U, Böhm M, Speck U: **Treatment of coronary in-stent restenosis with a paclitaxel-coated balloon catheter**. *The New England journal of medicine* 2006, **355**(20):2113-2124.

15. Unverdorben M, Vallbracht C, Cremers B, Heuer H, Hengstenberg C, Maikowski C, Werner GS, Antoni D, Kleber FX, Bocksch W *et al*: **Paclitaxel-coated balloon catheter versus paclitaxel-coated stent for the treatment of coronary in-stent restenosis**. *Circulation* 2009, **119**(23):2986-2994.

16. Rittger H, Brachmann J, Sinha AM, Waliszewski M, Ohlow M, Brugger A, Thiele H, Birkemeyer R, Kurowski V, Breithardt OA *et al*: **A randomized, multicenter, single-blinded trial comparing paclitaxel-coated balloon angioplasty with plain balloon angioplasty in drug-eluting stent restenosis: the PEPCAD-DES study**. *Journal of the American College of Cardiology* 2012, **59**(15):1377-1382.

17. Xu B, Gao R, Wang J, Yang Y, Chen S, Liu B, Chen F, Li Z, Han Y, Fu G *et al*: **A prospective, multicenter, randomized trial of paclitaxel-coated balloon versus paclitaxel-eluting stent for the treatment of drug-eluting stent in-stent restenosis: results from the PEPCAD China ISR trial**. *JACC Cardiovascular interventions* 2014, **7**(2):204-211.

18. Jensen CJ, Richardt G, Tölg R, Erglis A, Skurk C, Jung W, Neumann FJ, Stangl K, Brachmann J, Fischer D *et al*: **Angiographic and clinical performance of a paclitaxel-coated balloon compared to a second-generation sirolimus-eluting stent in patients with in-stent restenosis: the BIOLUX randomised controlled trial**. *EuroIntervention : journal of EuroPCR in collaboration with the Working Group on Interventional Cardiology of the European Society of Cardiology* 2018, **14**(10):1096-1103.

19. Scheller B, Hehrlein C, Bocksch W, Rutsch W, Haghi D, Dietz U, Böhm M, Speck U: **Two year follow-up after treatment of coronary in-stent restenosis with a paclitaxel-coated balloon catheter**. *Clinical research in cardiology : official journal of the German Cardiac Society* 2008, **97**(10):773-781.

20. Alfonso F, Pérez-Vizcayno MJ, Cárdenas A, García Del Blanco B, Seidelberger B, Iñiguez A, Gómez-Recio M, Masotti M, Velázquez MT, Sanchís J *et al*: **A randomized comparison of drug-eluting balloon versus everolimus-eluting stent in patients with bare-metal stent-in-stent restenosis: the RIBS V Clinical Trial (Restenosis Intra-stent of Bare Metal Stents: paclitaxel-eluting balloon vs. everolimus-eluting stent)**. *Journal of the American College of Cardiology* 2014, **63**(14):1378-1386.

21. Ali RM, Abdul Kader M, Wan Ahmad WA, Ong TK, Liew HB, Omar AF, Mahmood Zuhdi AS, Nuruddin AA, Schnorr B, Scheller B: **Treatment of Coronary Drug-Eluting Stent Restenosis by a Sirolimus- or Paclitaxel-Coated Balloon**. *JACC Cardiovascular interventions* 2019, **12**(6):558-566.

22. Cortese B, Micheli A, Picchi A, Coppolaro A, Bandinelli L, Severi S, Limbruno U: **Paclitaxel-coated balloon versus drug-eluting stent during PCI of small coronary vessels, a prospective randomised clinical trial. The PICCOLETO study**. *Heart (British Cardiac Society)* 2010, **96**(16):1291-1296.

23. Alfonso F, Pérez-Vizcayno MJ, Cárdenas A, García del Blanco B, García-Touchard A, López-Minguéz JR, Benedicto A, Masotti M, Zueco J, Iñiguez A *et al*: **A Prospective Randomized Trial of Drug-Eluting Balloons Versus Everolimus-Eluting Stents in Patients With In-Stent Restenosis of Drug-Eluting Stents: The RIBS IV Randomized Clinical Trial**. *Journal of the American College of Cardiology* 2015, **66**(1):23-33.

24. Aoki J, Nakazawa G, Ando K, Nakamura S, Tobaru T, Sakurada M, Okada H, Hibi K, Zen K, Habara S *et al*: **Effect of combination of non-slip element balloon and drug-coating balloon for in-stent restenosis lesions (ELEGANT study)**. *Journal of cardiology* 2019, **74**(5):436-442.

25. Naganuma T, Latib A, Sgueglia GA, Menozzi A, Castriota F, Micari A, Cremonesi A, De Felice F, Marchese A, Tespili M *et al*: **A 2-year follow-up of a randomized multicenter study comparing a paclitaxel drug-eluting balloon with a paclitaxel-eluting stent in small coronary vessels the BELLO study**. *International journal of cardiology* 2015, **184**:17-21.

26. Habara S, Iwabuchi M, Inoue N, Nakamura S, Asano R, Nanto S, Hayashi Y, Shiode N, Saito S, Ikari Y *et al*: **A multicenter randomized comparison of paclitaxel-coated balloon catheter with conventional balloon angioplasty in patients with bare-metal stent restenosis and drug-eluting stent restenosis**. *American heart journal* 2013, **166**(3):527-533.

27. Jing QM, Zhao X, Han YL, Gao LL, Zheng Y, Li ZQ, Yang P, Cong HL, Gao CY, Jiang TM *et al*: **A drug-eluting Balloon for the trEatment of coronarY bifurcatiON lesions in the side branch: a prospective multicenter ranDomized (BEYOND) clinical trial in China**. *Chinese medical journal* 2020, **133**(8):899-908.

28. Zhu J, Liu L, Zhu Z, Yang Z, Hu J, Ding F, Zhou Y, Su X, Ge J, Liu X *et al*: **A randomized comparison of a novel iopromide-based paclitaxel-coated balloon Shenqi versus SeQuent Please for the treatment of in-stent restenosis**. *Coronary artery disease* 2021, **32**(6):526-533.

29. Hu P, Sun Y, Li CL, Jin R, Xie Q, Jiang XJ, Wu LP, Jiang JJ, Qiu XB, Cao Y *et al*: **A randomized comparison of two paclitaxel-coated balloons for the treatment of in-stent restenosis: The LONGTY ISR China randomized trial (LONGTY DCB vs. SeQuent Please DCB)**. 2021, **97 Suppl 2**:988-995.

30. Belkacemi A, Agostoni P, Voskuil M, Stella PR: **Coronary bifurcation lesions treated with the drug-eluting balloon: a preliminary insight from the DEBIUT study**. *EuroIntervention : journal of EuroPCR in collaboration with the Working Group on Interventional Cardiology of the European Society of Cardiology* 2011, **7 Suppl K**:K66-69.

31. Hamm CW, Dörr O, Woehrle J, Krackhardt F, Ince H, Zeus T, Berland J, Piot C, Roubille F, Schult I *et al*: **A multicentre, randomised controlled clinical study of drug-coated balloons for the treatment of coronary in-stent restenosis**. *EuroIntervention : journal of EuroPCR in collaboration with the Working Group on Interventional Cardiology of the European Society of Cardiology* 2020, **16**(4):e328-e334.

32. Funatsu A, Nakamura S, Inoue N, Nanto S, Nakamura M, Iwabuchi M, Ando K, Asano R, Habara S, Saito S *et al*: **A multicenter randomized comparison of paclitaxel-coated balloon with plain balloon angioplasty in patients with small vessel disease**. *Clinical research in cardiology : official journal of the German Cardiac Society* 2017, **106**(10):824-832.

33. Ali RM, Degenhardt R, Zambahari R, Tresukosol D, Ahmad WA, Kamar H, Kui-Hian S, Ong TK, bin Ismail O, bin Elis S *et al*: **Paclitaxel-eluting balloon angioplasty and cobalt-chromium stents versus conventional angioplasty and paclitaxel-eluting stents in the treatment of native coronary artery stenoses in patients with diabetes mellitus**. *EuroIntervention : journal of EuroPCR in collaboration with the Working Group on Interventional Cardiology of the European Society of Cardiology* 2011, **7 Suppl K**:K83-92.

34. Hao X, Huang D, Wang Z, Zhang J, Liu H, Lu Y: **Study on the safety and effectiveness of drug-coated balloons in patients with acute myocardial infarction**. *Journal of cardiothoracic surgery* 2021, **16**(1):178.

35. Vos NS, van der Schaaf RJ, Amoroso G, Herrman JP, Patterson MS, Slagboom T, Vink MA: **REVascularization with paclitaxEL-coated balloon angioplasty versus drug-eluting stenting in acute myocardial infarcTION-A randomized controlled trial: Rationale and design of the REVELATION trial**. *Catheterization and cardiovascular interventions : official journal of the Society for Cardiac Angiography & Interventions* 2016, **87**(7):1213-1221.

36. Gobić D, Tomulić V, Lulić D, Židan D, Brusich S, Jakljević T, Zaputović L: **Drug-Coated Balloon Versus Drug-Eluting Stent in Primary Percutaneous Coronary Intervention: A Feasibility Study**. *The American journal of the medical sciences* 2017, **354**(6):553-560.

37. Scheller B, Ohlow MA, Ewen S, Kische S, Rudolph TK, Clever YP, Wagner A, Richter S, El-Garhy M, Böhm M *et al*: **Bare metal or drug-eluting stent versus drug-coated balloon in non-ST-elevation myocardial infarction: the randomised PEPCAD NSTEMI trial**. *EuroIntervention : journal of EuroPCR in collaboration with the Working Group on Interventional Cardiology of the European Society of Cardiology* 2020, **15**(17):1527-1533.

38. Yu X, Wang X, Ji F: **A Non-inferiority, Randomized Clinical Trial Comparing Paclitaxel-Coated Balloon Versus New-Generation Drug-Eluting Stents on Angiographic Outcomes for Coronary De Novo Lesions**. *Cardiovascular Drugs and Therapy* 2022, **36**(4):655-664.

39. Qian J, Wu Y, Li C, Yin J, Fu G, Wang J, He Y, Ma G, Chen Y: **Drug-coated balloon for the treatment of small vessel disease: 9 months of angiographic results and 12 months of clinical outcomes of the PEPCAD China SVD study**. *Catheterization and Cardiovascular Interventions* 2023, **101**(1):33-43.

40. Liu S, Zhou Y, Shen Z, Chen H, Qiu C, Fu G, Li H, Yu Z, Zeng Q, Li Z *et al*: **A Randomized Comparison of 2 Different Drug-Coated Balloons for In-Stent Restenosis**. *JACC Cardiovasc Interv* 2023, **16**(7):759-767.

41. Ahmad WAW, Nuruddin AA, Abdul Kader M, Ong TK, Liew HB, Ali RM, Mahmood Zuhdi AS, Ismail MD, Yusof AKM, Schwenke C *et al*: **Treatment of Coronary De Novo Lesions by a Sirolimus- or Paclitaxel-Coated Balloon**. *JACC Cardiovasc Interv* 2022, **15**(7):770-779.

42. Hu P, Sun Y, Li CL, Jin R, Xie Q, Jiang XJ, Wu LP, Jiang JJ, Qiu XB, Cao Y *et al*: **A randomized comparison of two paclitaxel-coated balloons for the treatment of in-stent restenosis: The LONGTY ISR China randomized trial (LONGTY DCB vs. SeQuent Please DCB)**. *Catheterization and Cardiovascular Interventions* 2021, **97 Suppl 2**:988-995.

43. Xu K, Fu G, Tong Q, Liu B, Han X, Zhang J, Ma G, Yang Q, Li H, Zhou Y *et al*: **Biolimus-Coated Balloon in Small-Vessel Coronary Artery Disease: The BIO-RISE CHINA Study**. *JACC Cardiovasc Interv* 2022, **15**(12):1219-1226.

44. Nakamura M, Isawa T, Nakamura S, Ando K, Namiki A, Shibata Y, Shinke T, Ito Y, Fujii K, Shite J *et al*: **Drug-Coated Balloon for the Treatment of Small Vessel Coronary Artery Disease　- A Randomized Non-Inferiority Trial**. *Circulation journal : official journal of the Japanese Circulation Society* 2023, **87**(2):287-295.

45. Wang Z, Yin Y, Li J, Qi W, Yu B, Xu Z, Zhu W, Yang F, Cao M, Zhang H: **New Ultrasound-Controlled Paclitaxel Releasing Balloon vs. Asymmetric Drug-Eluting Stent in Primary ST-Segment Elevation Myocardial Infarction　- A Prospective Randomized Trial**. *Circulation journal : official journal of the Japanese Circulation Society* 2022, **86**(4):642-650.

46. Valgimigli M, Bueno H, Byrne RA, Collet JP, Costa F, Jeppsson A, Juni P, Kastrati A, Kolh P, Mauri L *et al*: **2017 ESC focused update on dual antiplatelet therapy in coronary artery disease developed in collaboration with EACTS: The Task Force for dual antiplatelet therapy in coronary artery disease of the European Society of Cardiology (ESC) and of the European Association for Cardio-Thoracic Surgery (EACTS)**. *Eur Heart J* 2018, **39**(3):213-260.
